# Pathways Linking Parental Social Support and Decision-Making Participation to Medication Adherence in Children With Epilepsy: The Moderating Role of Parental Anxiety

**DOI:** 10.1155/da/7159579

**Published:** 2025-09-16

**Authors:** Chunsong Yang, Rui Huang, Qiuji Tao, Zilong Hao, Li Zhao, Lingli Zhang

**Affiliations:** ^1^Department of Pharmacy, Evidence-Based Pharmacy Center, West China Second Hospital, Sichuan University, Chengdu, China; ^2^Key Laboratory of Birth Defects and Related Diseases of Women and Children (Sichuan University), Ministry of Education, Chengdu, China; ^3^School of Nursing, Southwest Medical University, Luzhou, China; ^4^Nursing Unit of Pediatric Neurology, West China Second University Hospital, Sichuan University, Chengdu, China; ^5^Department of Neurology, West China Hospital, Sichuan University, Chengdu, China; ^6^Department of Health Policy and Management, West China School of Public Health/West China Fourth Hospital, Sichuan University, Chengdu, China

**Keywords:** medication adherence, parental anxiety, parental decision-making, pediatric epilepsy, social support

## Abstract

**Background:** Medication adherence among pediatric epilepsy patients is frequently suboptimal, and the complex interplay between parental social support, decision-making participation, treatment satisfaction, and parental anxiety in influencing medication adherence remains underexplored. This study investigates both the direct and indirect pathways linking these factors to medication adherence and examines the mediating role of treatment satisfaction and the moderating role of parental anxiety.

**Methods:** A cross-sectional study was conducted at three medical institutions between January 2020 and June 2024. Data on patient demographics and standardized scales measuring medication adherence, social support, communication and decision-making participation, treatment satisfaction, and parental anxiety were collected. Relationships among these variables were analyzed using structural equation modeling (SEM) and moderation analysis.

**Results:** A total of 1056 patients were included in the study, with a mean age of 8.86 ± 3.99 years; 51.7% were male. Path analysis showed that parental social support (STD = 0.344, *p*  < 0.001), communication and decision-making participation (STD = 0.392, *p*  < 0.001), and treatment satisfaction (STD = 0.090, *p*  < 0.05) had significant positive effects on medication adherence. Parental social support (STD = 0.483, *p*  < 0.001) and communication and decision-making participation (STD = 0.203, *p*  < 0.001) also strongly influenced treatment satisfaction. The indirect effects of social support and decision-making participation on medication adherence, mediated through treatment satisfaction, were statistically significant (*p*  < 0.05). Parental anxiety, as a moderating factor, weakened the positive effects of social support, decision-making participation, and treatment satisfaction on medication adherence (*p*  < 0.05).

**Conclusion:** This study systematically develops an integrated model linking parental social support, communication and decision-making participation, treatment satisfaction, and anxiety to medication adherence in pediatric epilepsy. It highlights the mediating role of treatment satisfaction and the moderating role of parental anxiety. Enhancing parental social support and communication, improving treatment satisfaction, and addressing parental anxiety are key strategies to promote medication adherence.

## 1. Introduction

### 1.1. Epidemiological Status

Epilepsy is a common neurological disorder marked by recurrent seizures caused by abnormal neuronal discharges in the brain. It ranks among the leading causes of disability and mortality worldwide [1]. Globally, around 65 million people suffer from epilepsy, with a prevalence of 5–10 per 1000 children in developed countries. However, this rate is significantly higher in developing countries, ranging from 10 to 20 per 1000 children [2, 3]. In China, over 1 million children are affected by epilepsy [4]. For children, who are in critical stages of physiological, psychological, and social development, epilepsy's impact extends beyond physical health, affecting cognitive development, mental well-being, and social adaptability [1, 5].

### 1.2. Current Status and Issues in Medication Adherence

Long-term antiseizure medications (ASMs) are the primary treatment for epilepsy, with adherence being critical for achieving effective outcomes [6]. In this study, “medication adherence” refers to the extent to which pediatric epilepsy patients take ASMs as prescribed by healthcare providers, including correct dosage, timing, and frequency over a sustained period. While some children may require lifelong ASM treatment, others may have their dosages adjusted or withdrawn under medical supervision based on seizure control and developmental progress. However, adherence rates among pediatric epilepsy patients remain low, at approximately 50%–60% globally [6]. In low- and middle-income countries, these rates drop further to 30%–40% [7]. Poor adherence results in inadequate seizure control, increased recurrence risks, more complications, higher hospitalization costs, and even greater mortality [1, 8].

While studies have examined factors influencing medication adherence in pediatric epilepsy, several limitations persist. Most research primarily focuses on medication-related factors (e.g., dosage, side effects) and healthcare system issues (e.g., service accessibility). Although these factors are important, previous studies have employed various methods to assess medication adherence, including parent-reported questionnaires, clinician assessments, pill counts, and electronic monitoring devices [9, 10]. Currently, there is no universally accepted “gold standard” for adherence measurement. Among existing methods, parent-reported questionnaires remain the most commonly used due to their cost-effectiveness and feasibility in large-scale studies [11–13]. Despite their subjective nature, they have demonstrated moderate to high correlations with objective measures such as electronic medication event monitoring systems (MEMSs), making them a practical component in adherence assessment [6, 13].

Although these studies have provided useful insights, there is limited research on psychosocial factors. Psychosocial factors, such as perceived social support, caregiver involvement, and anxiety, are believed to influence adherence through mechanisms like enhancing motivation, improving understanding of treatment, and reducing psychological resistance [14–16]. In other chronic pediatric illnesses such as diabetes and asthma, research has shown that higher caregiver support and lower anxiety levels are associated with better adherence outcomes [6, 17–19]. These findings suggest the relevance of psychosocial dynamics in epilepsy treatment as well. However, few comprehensive multidimensional models have been developed. Current studies often focus on single variables, overlooking the interactions and dynamic mechanisms among multiple factors.

Moreover, research on social support remains insufficient. While social support is widely recognized as a protective factor in chronic disease management, existing studies mainly address its direct effects, neglecting potential mediating pathways, such as its role in enhancing treatment satisfaction [6, 20]. This gap results in a fragmented understanding of the actual mechanisms underlying social support. Similarly, studies on caregiver participation in treatment decision-making lack depth and breadth. Preliminary evidence suggests that caregiver involvement improves adherence, but most studies focus on single variables or qualitative analyses, failing to reveal the interactions between caregiver participation, social support, and treatment satisfaction [6, 21, 22].

Caregiver anxiety has also been identified as a critical but underexplored factor. While it is known that anxiety can impair decision-making and disease management capacity, its specific manifestations and interactions with other psychosocial factors in the context of epilepsy remain unclear [23]. In other chronic conditions, such as diabetes and asthma [17–19], caregiver anxiety has been shown to interfere with medication routines and reduce adherence by creating emotional and cognitive barriers [24, 25]. Anxious caregivers may feel overwhelmed due to emotional stress, leading them to overlook or miss medication administration times. Additionally, anxiety can affect the caregiver's attention and memory, making it difficult for them to administer medication on time or follow complex medication regimens. Anxious caregivers may also be reluctant to communicate with healthcare professionals, which can result in missed opportunities to adjust the treatment plan, further reducing medication adherence [24, 25]. These findings form the theoretical basis for investigating the moderating role of caregiver anxiety in ASM adherence in pediatric epilepsy.

Finally, the moderating effect of caregiver anxiety on adherence has not been systematically explored. While caregiver anxiety is known to impair decision-making and disease management, its specific manifestations and interactions with other factors remain unclear, limiting the development of targeted interventions [26, 27].

### 1.3. Theoretical Framework

#### 1.3.1. The Protective Role of Caregiver Social Support

Social support is a critical protective factor in chronic disease management, significantly influencing the psychological well-being and disease management capacity of patients and their families [28]. For children with epilepsy, social support encompasses emotional support (e.g., encouragement from family and friends), informational support (e.g., disease management guidance from healthcare providers), and practical support (e.g., financial assistance) [6, 28]. High levels of social support among caregivers improve medication adherence and alleviate psychological stress [29], thereby enhancing trust in healthcare services and boosting treatment satisfaction.

#### 1.3.2. The Importance of Caregiver Participation in Decision-Making

Caregivers play a central role in the treatment of pediatric epilepsy. Their active involvement in communicating with healthcare providers, developing treatment plans, and supervising medication use significantly impacts treatment outcomes [22, 30]. Research shows that children of caregivers who actively participate in treatment decisions exhibit higher adherence rates than those whose caregivers are less involved [31]. Moreover, caregiver participation enhances treatment satisfaction, further contributing to improved adherence [32].

#### 1.3.3. The Impact of Treatment Satisfaction on Adherence

Treatment satisfaction reflects patients' overall evaluation of treatment outcomes, physician-patient communication, service attitudes, and economic costs. It not only represents subjective experiences but also significantly influences adherence, which is essential for long-term treatment success. While existing studies confirm the positive impact of treatment satisfaction on adherence [33], research focusing on pediatric epilepsy is limited, particularly regarding its mediating role in the relationship between social support, caregiver participation, and adherence.

#### 1.3.4. The Moderating Effect of Caregiver Anxiety

The unpredictable and long-term nature of epilepsy treatment often leads to caregiver anxiety, which may weaken their ability to manage the disease effectively. Approximately 25.7% of caregivers of children with epilepsy experience moderate to severe anxiety [34]. Caregiver anxiety can reduce the positive effects of social support, impair decision-making ability, and diminish management efficiency, ultimately lowering medication adherence [18, 19, 35].

Taken together, existing research highlights the importance of social support, caregiver participation, treatment satisfaction, and caregiver anxiety in shaping medication adherence in chronic pediatric conditions. However, few studies have systematically examined these factors in an integrated framework within the context of pediatric epilepsy. In particular, the complex interplay among caregiver-perceived social support, participation in treatment decision-making, treatment satisfaction, and anxiety remains insufficiently understood. To address these gaps, this study aims to investigate the direct and indirect pathways through which these psychosocial variables influence medication adherence among children with epilepsy. Specifically, it explores the mediating role of treatment satisfaction and the moderating role of caregiver anxiety, thereby offering a more comprehensive understanding of adherence mechanisms and informing targeted intervention strategies.

## 2. Materials and Methods

### 2.1. Study Design

**Figure 1:**
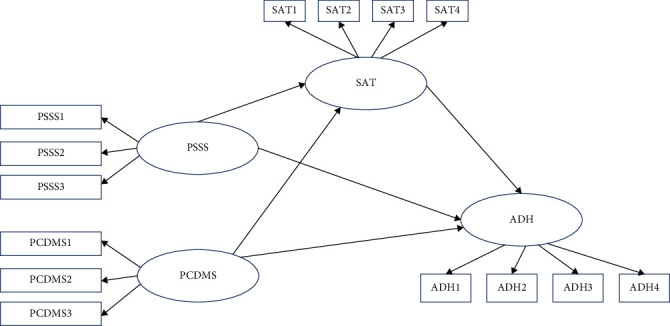
Theoretical hypothesis path diagram.

This cross-sectional quantitative study aimed to explore how factors such as social support, communication and decision-making participation, treatment satisfaction, and caregiver anxiety influence medication adherence in children with epilepsy. A path model was developed to analyze these factors. A hypothesized path model was constructed based on existing literature and theoretical assumptions in the introduction. The model posits both direct and indirect effects among psychosocial variables, with medication adherence as the primary outcome variable. The hypothesized relationships were tested using structural equation modeling (SEM), which allows for simultaneous analysis of multiple mediating and moderating effects. The following path model was proposed (Figure 1): (1) caregiver social support has a direct positive impact on medication adherence. (2) Caregiver communication and decision-making participation have a direct positive impact on medication adherence. (3) Caregiver treatment satisfaction has a direct positive impact on medication adherence. (4) Caregiver social support indirectly affects medication adherence through treatment satisfaction. (5) Caregiver communication and decision-making participation indirectly affect medication adherence through treatment satisfaction. (6) Caregiver anxiety negatively moderates the effect of social support on medication adherence. (7) Caregiver anxiety negatively moderates the effect of communication and decision-making participation on medication adherence. (8) Caregiver anxiety negatively moderates the effect of treatment satisfaction on medication adherence.

### 2.2. Study Population

The study included children with epilepsy and their caregivers who attended three healthcare facilities (the Jinjiang and Huaxi campuses of West China Second University Hospital and the Sichuan Children's Hospital) between January 2020 and June 2024. West China Second Hospital is a women's and children's specialty hospital under the budget management of the National Health Commission. It serves as the National Regional Medical Center for Children (Southwest China) and is the largest women's and children's hospital in Western China. The hospital provides services to 3.521 million outpatient and emergency visits annually, and discharges 100,000 patients each year.

#### 2.2.1. Inclusion Criteria

(1) Children must have a confirmed diagnosis of epilepsy based on International League Against Epilepsy (ILAE) criteria and have been receiving antiepileptic drug treatment for at least 3 months; (2) children aged 0–18 years; and (3) caregivers able to complete the questionnaire and provide informed consent.

#### 2.2.2. Exclusion Criteria

(1) Children with severe mental disorders or developmental issues (e.g., autism spectrum disorder, cerebral palsy, or an intelligence quotient < 70), or with chronic physical conditions lasting more than 3 months (e.g., diabetes, asthma, or congenital heart disease). (2) Caregivers with communication difficulties or those unwilling to participate.

### 2.3. Data Collection

Data collection involved obtaining baseline demographic information and administering multiple measurement scales during the participants' first outpatient visit. Trained investigators conducted face-to-face data collection in designated consultation rooms or waiting areas. Caregivers completed standardized questionnaires with assistance from the investigators to ensure comprehension and minimize missing data. Medical records were also reviewed to supplement the information. All completed questionnaires were reviewed on-site for completeness and clarity. Subsequently, the data were double-entered into a secure database and independently verified by two researchers to ensure accuracy and consistency. Any discrepancies were resolved by cross-checking with the original records. This study was conducted prospectively and adopted a cross-sectional survey design.

#### 2.3.1. Baseline Data Collection

The epilepsy patients we recruited were divided into two groups: one group consisted of patients with a previous diagnosis (follow-up patients), and the other group included newly diagnosed patients. The epilepsy diagnosis for all patients was confirmed based on the diagnostic data from the medical system. For follow-up patients who were already diagnosed and receiving pharmacotherapy, we confirmed their medication history through medical records. If their medication history met the requirement of at least 3 months, they were included in the study. For newly diagnosed patients, we began pharmacotherapy after their diagnosis. We will conduct follow-up assessments after 3 months of treatment. During this phase, we will evaluate their medication adherence to ensure it meets the study criteria. Therefore, the research team collected the basic characteristics of the children and their families using a standardized questionnaire. The baseline information included the patients' age, sex, newly diagnosed status, seizure type, comorbidities, family history, whether the patient was an only child, the relationship of the guardian, the guardian's occupational status, and the guardian's age.

## 3. Measurement Scales

### 3.1. Communication and Decision-Making Scale (CDMS)

A self-designed scale included three dimensions: information transmission and transparency; decision-making participation and negotiation; and personalization and patient care. The scale was developed following a multistep process: (1) item generation was informed by a literature review and semi-structured interviews with five caregivers and five pediatric neurologists; (2) items were reviewed by a panel of experts in pediatric neurology and health communication to assess content validity; (3) cognitive pretesting was conducted with 10 caregivers to evaluate item clarity and relevance. Revisions were made accordingly, and the finalized scale was used in the study.

Three dimensions include the following nine items.

#### 3.1.1. Dimension 1

Information transmission and transparency (3 items): Did the medical staff provide clear information and the best treatment plan? Was the purpose of the medication fully explained? Did the medical staff communicate transparently without withholding information about the condition?

#### 3.1.2. Dimension 2

Decision-making participation and negotiation (3 items): Did the medical staff seek the patient's opinion on the treatment? Does the patient have a need to participate in treatment decision-making? Was patient consent obtained before clinical examinations?

#### 3.1.3. Dimension 3

Personalization and patient care (3 items): Did the medical staff help reduce the psychological stress of the patient or their child? Was the medical staff familiar with the patient's condition and able to provide assistance? Did the medical staff offer personalized care?

Each item was scored on a 5-point Likert scale ranging from 1 (strongly disagree) to 5 (strongly agree). Higher scores indicated better communication and greater caregiver satisfaction with decision-making participation.

### 3.2. Adherence to Medication Scale (ADH)

A self-designed scale consisting of 4 items: “Have you ever forgotten to administer medication?”, “Do you sometimes neglect administering medication?”, “Have you ever stopped administering medication when symptoms improved?”, “Have you ever stopped administering medication when symptoms worsened?”

The development process of the scale was the same as that of the CDMS. A “yes” response was scored as 0, and a “no” as 1. Total scores ranged from 0 to 4, with scores of 4 indicating high adherence and ≤3 indicating low adherence. The ADH assesses medication adherence over the past 3 months.

### 3.3. Perceived Social Support Scale (PSSS)

The PSSS was developed to assess individuals' perceived support from three sources: family, friends, and significant others. This scale has demonstrated good psychometric properties across a wide range of populations, including adolescents, adults, and caregivers in various cultural contexts. Previous studies have validated the PSSS in both clinical and community samples, showing strong internal consistency and construct validity [36]. In this study, the PSSS was used to measure caregivers' perceived levels of social support, which is a key psychosocial factor influencing medication adherence and mental health outcomes [36, 37].

This scale consists of 12 items assessing three dimensions of social support: family support, friend support, and other support [38]. Each item was rated on a 7-point Likert scale (1 = strongly disagree, 7 = strongly agree), with total scores ranging from 12 to 84. Scores of 12–36 reflect low support, 37–60 moderate support, and 61–84 high support. Higher scores indicated greater perceived social support.

### 3.4. Generalized Anxiety Disorder-7 (GAD-7) Scale

The GAD-7 is a brief self-report instrument developed to screen for symptoms of generalized anxiety disorder. It consists of seven items based on DSM-IV criteria and has been widely validated in general and clinical populations [39, 40]. The GAD-7 has shown high internal consistency and strong criterion validity when compared with structured psychiatric interviews. It is frequently used in both research and clinical settings to evaluate anxiety severity [39, 40]. In the context of this study, the GAD-7 was employed to assess anxiety symptoms among caregivers, given that caregiver psychological distress can impact decision-making, communication, and treatment adherence [40].

This scale measures caregivers' levels of generalized anxiety [40]. It consists of seven items, each rated on a 4-point Likert scale (0 = not at all, 1 = several days, 2 = more than half the days, 3 = nearly every day), with total scores ranging from 0 to 21. Scores were interpreted as follows: 0–4 = no anxiety and > 5 = anxiety.

### 3.5. Treatment Satisfaction Scale (SAT)

A self-designed scale consisting of 4 items: “I am satisfied with the current treatment plan,” “I am satisfied with the healthcare providers' service attitude,” “I am satisfied with the healthcare providers' medical expertise,” and “I am satisfied with the hospital's medical procedures.”

The development process of the scale was the same as that of the CDMS. Each item was scored on a 5-point Likert scale (1 = very dissatisfied, 5 = very satisfied). Higher scores indicated greater caregiver satisfaction with treatment.

### 3.6. Sample Size Estimation

Sample size estimation considered both cross-sectional survey requirements and SEM. We used the formula (*n* = *u*^2^*π*(1 − *π*)/*δ*^2^) for the estimation of sample size when estimating the population rate in the sampling survey to calculate the minimum patient sample size. *n* is the required sample size, *π* represents the estimated proportion of the population with good medication adherence, *δ* is the permissible margin of error, and *u* is the *z*-score corresponding to the desired confidence level (1.96 for 95% confidence). Based on previous research, we used an estimated adherence rate of 58% (*π* = 0.58) [6]. Assuming a 95% confidence level (*u* = 1.96) and a margin of error of 0.03 (*δ* = 0.03), the calculated minimum required sample size was: *n* = (1.96^2^ × 0.58 × 0.42) /0.03^2^, ≈ 1040 participants. For SEM, the sample size should be 10–20 times the number of model variables. Since this study involved eight pathways and five main predictive variables, an estimated sample size of 200–300 participants was needed. To ensure robustness, the study adopted the higher estimate of 1040 participants.

## 4. Quality Control

This study implemented several quality control measures to ensure the scientific rigor and reliability of the data. First, the questionnaire was developed based on established theoretical models and was subjected to expert review and pilot testing with a small sample to verify its reliability and validity. The reliability and validity of the scale were evaluated using multiple methods. Internal consistency reliability was assessed using Cronbach's alpha coefficient, while content validity was established through expert review by a panel of specialists in pediatric neurology and health communication, who evaluated the relevance, clarity, and representativeness of all items. Prior to data collection, all researchers received standardized training to ensure familiarity with the questionnaire content and standardized procedures, thereby maintaining consistency and compliance throughout the data collection process. During the data collection phase, guardians completed the questionnaires on-site, with the research team providing continuous guidance. Upon completion, the questionnaires were thoroughly reviewed, and invalid responses were excluded. All data were independently entered by two researchers, who then cross-checked and verified the entries.

## 5. Data Analysis

Data analysis was conducted using SPSS 26.0 and AMOS 24.0 software, following these steps: (1) descriptive statistics: summarized baseline data, such as child characteristics and family features. (2) Reliability analysis: assessed internal consistency of the scales using Cronbach's *α* coefficient. (3) Validity analysis: conducted KMO and Bartlett's tests to confirm data suitability, followed by exploratory and confirmatory factor analyses to evaluate scale validity. (4) Correlation analysis: examined relationships between medication adherence scores and scores for treatment satisfaction, social support, communication and decision-making participation, and anxiety using Pearson correlation. (5) Path analysis: used SEM to test hypothesized pathways and assess the direct and indirect effects of caregiver social support, communication and decision-making participation, treatment satisfaction, and caregiver anxiety on medication adherence. (6) Moderating effect analysis: based on previous literature [41], age, whether the child is an only child, the guardian's occupation, and comorbidities were selected as control variables. The analysis aimed to examine the pathways through which social support, communication and decision-making participation, and treatment satisfaction influence medication adherence, while also testing the moderating role of the guardian's anxiety level in these relationships.

## 6. Ethical Considerations

This study followed the principles of the Declaration of Helsinki and was approved by the Ethics Committee of West China Second University Hospital (Ethics Approval Number 2019007), Sichuan University. Prior to participation, informed consent was obtained from the parents or legal guardians of all participants under the age of 18. In cases where the accompanying person was not a legal guardian but a caregiver (e.g., grandparent or other family member), informed consent was still obtained from the legal guardian, either on-site or through written authorization, in accordance with ethical requirements. The research team provided a detailed explanation of the study purpose, data confidentiality measures, and potential risks to the caregivers present. Only after this explanation were consent forms signed by the legal guardians. This procedure ensured that all participants aged 0–18 years were included in the study with valid informed consent from legally authorized representatives.

## 7. Results

### 7.1. Characteristics of Included Patients (Table 1)

A total of 1128 patients were initially recruited for the study. Among these, 42 patients were not on medication, and 30 caregivers declined to participate. Ultimately, 1056 pediatric epilepsy patients were analyzed. The mean age of the patients was 8.86 ± 3.99 years, with males accounting for 51.7% (546/1056). Of the total cohort, 50.5% (533/1056) were newly diagnosed cases, and 7.1% (75/1056) had a family history of epilepsy. Regarding seizure types, focal/partial seizures were the most prevalent, observed in 45.8% (484 cases), followed by generalized seizures in 38.4% (405 cases). Patients exhibiting both generalized and focal seizures accounted for 11.6% (123 cases), while 4.1% (44 cases) had unclassified seizure types.

The proportion of patients who were only children was 43.9% (464/1056). Comorbidities were identified in 57.1% (603/1056) of the patients, including developmental delay in 22.2% (235 cases), migraine in 17.3% (183 cases), sleep disorders in 8.3% (88 cases), tic disorders in 3.4% (36 cases), attention-deficit hyperactivity disorder (ADHD) in 2.6% (27 cases), intellectual disability in 2.2% (23 cases), hyperactivity disorder in 0.5% (5 cases), depression or anxiety in 0.4% (4 cases), and autism in 0.2% (2 cases).

The mean scores of ADH, SAT, PSSS, CDMS, and ANX were 2.75 ± 1.691, 14.77 ± 3.649, 52.907 ± 14.974, 30.552 ± 6.86, and 5.666 ± 6.386, respectively. A total of 38.7% (409/1056) of the patients exhibited poor treatment adherence, 12.9% (136/1056) of the caregivers had low social support, 56.6% (598/1056) had moderate social support, and 30.5% (322/1056) had high social support.

### 7.2. Reliability Analysis

Internal consistency was good across all scales, with Cronbach's *α* ranging from 0.803 to 0.942 (all > 0.7). Details are provided in Appendix Table A1.

### 7.3. Validity Analysis

#### 7.3.1. Exploratory Factor Analysis

The KMO value was 0.915, and Bartlett's test was significant (*χ*^2^ = 28,634.016, *p* < 0.001), confirming suitability for factor analysis. Seven principal factors explained 78.716% of the variance, with all factor loadings > 0.4, indicating strong structural validity. Details are provided in Appendix Tables A2–A4.

#### 7.3.2. Confirmatory Factor Analysis

CFA showed good model fit (CMIN/DF = 1.971, GFI = 0.945, TLI = 0.978, CFI = 0.981, RMSEA = 0.030). Factor loadings ranged from 0.7 to 0.92. All constructs showed CR > 0.7 and AVE > 0.5, confirming convergent validity. Discriminant validity was supported as each AVE square root exceeded interconstruct correlations. Details are provided in Appendix TablesA5–A8.

### 7.4. Correlation Analysis

Medication adherence was significantly positively correlated with satisfaction (*r* = 0.390), social support (*r* = 0.446), and communication/decision-making (*r* = 0.465) (all *p* < 0.01). No significant correlation was found with caregiver anxiety (*r* = −0.058, *p* > 0.05), suggesting potential indirect effects. Details are provided in Appendix Table A9.

### 7.5. Structural Equation Model Analysis

Path relationships were analyzed using a structural equation model; the details are provided in Appendix Table A10. The fit indices indicated good model fit (e.g., CMIN/DF = 3.205, CFI = 0.982, and RMSEA = 0.046). SEM confirmed that social support and communication and decision-making participation significantly improved medication adherence through both direct and indirect pathways. Specifically, path analysis results (Table 2) revealed that social support significantly promoted satisfaction (standardized path coefficient = 0.483, *p* < 0.001), as did communication and decision-making participation (standardized path coefficient = 0.203, *p* < 0.001).

Regarding medication adherence, both social support (standardized path coefficient = 0.344, *p* < 0.001) and communication and decision-making participation (standardized path coefficient = 0.392, *p* < 0.001) had significant direct positive effects. Additionally, satisfaction significantly promoted medication adherence (standardized path coefficient = 0.090, *p* < 0.05). These findings confirm the study's hypotheses, demonstrating that social support and communication and decision-making participation enhance medication adherence in pediatric epilepsy patients through both direct and indirect pathways.

### 7.6. Mediation Effect Analysis

Mediation effects were tested using SEM (Table 3). Results showed that treatment satisfaction partially mediated the relationships between social support, communication and decision-making participation, and medication adherence. Specifically, social support indirectly enhanced medication adherence through increased satisfaction, and this indirect effect was significant (*p*=0.018). The total effect, encompassing both direct and indirect effects, was positive. Similarly, communication and decision-making participation indirectly influenced medication adherence via improved satisfaction, with the indirect effect also being significant (*p*=0.018). The total effect remained positive.

### 7.7. Moderating Effect Analysis

Interaction analysis indicated that caregiver anxiety significantly negatively moderated the relationships between social support (*t* = −5.590, *p* < 0.05), communication and decision-making participation (*t* = −4.977, *p* < 0.05), and treatment satisfaction (*t* = −5.261, *p* < 0.05) with medication adherence (Tables 4–6). Specifically, higher caregiver anxiety levels weakened the positive effects of social support, communication and decision-making participation, and satisfaction on medication adherence. As caregiver anxiety increased, the positive factors influencing adherence became less effective and, in some cases, were partially offset.

## 8. Discussion

### 8.1. Main Findings of the Study

This study adopts a cross-sectional design to construct a comprehensive theoretical model that explores how social and psychological factors influence medication adherence in pediatric epilepsy through multiple pathways. For the first time, it systematically investigates the direct and indirect effects of social support and caregiver participation in decision-making on adherence while revealing the mediating role of treatment satisfaction and the moderating effect of caregiver anxiety. By deepening the understanding of the mechanisms influencing medication adherence, this research aims to provide scientific evidence for developing multilevel, personalized intervention strategies. The main findings are: (1) caregiver social support and communication and decision-making participation directly improved medication adherence and also indirectly influenced it through treatment satisfaction, showing that treatment satisfaction partially mediates these relationships. (2) Caregiver anxiety significantly moderated the relationships between these variables and medication adherence. High anxiety levels weakened the positive effects of social support, communication and decision-making participation, and treatment satisfaction. (3) The total effects of social support and caregiver communication and decision-making participation on adherence were significant. These findings emphasize the need for strategies to enhance social support and caregiver participation while addressing caregiver anxiety to optimize outcomes. By combining mediation and moderation analyses, this study provides new theoretical insights into the mechanisms of medication adherence in pediatric epilepsy patients.

### 8.2. Interpretation of Results

The positive effect of social support on medication adherence highlights the importance of a supportive environment in reducing caregivers' burden and enhancing their trust in and adherence to treatment plans. Social support enhances emotional security and confidence through the mediating effect of treatment satisfaction, improving caregivers' ability to adhere to medication regimens. This finding aligns with theories on the protective role of social support in chronic disease management [19, 20, 42, 43] and highlights the complex pathways identified through mediation analysis.

Similarly, caregiver communication and decision-making participation play a crucial role in improving adherence. Active caregiver involvement in treatment not only enhances their sense of ownership and recognition of treatment plans but also indirectly improves adherence through increased satisfaction. This result aligns with the concept of “shared decision-making,” [44, 45] which emphasizes the importance of trust and collaboration between healthcare providers and caregivers.

Treatment satisfaction serves as a central mediating variable in the medication adherence model, acting as a bridge between psychosocial factors and behavioral outcomes. However, caregiver anxiety significantly moderates this pathway, such that higher levels of anxiety diminish the positive effect of satisfaction on adherence. This attenuation may not simply reflect excessive worry, but rather a deeper cognitive and emotional overload caused by the long-term, unpredictable, and potentially stigmatizing nature of epilepsy. In the case of pediatric epilepsy, caregivers often face ongoing fears of breakthrough seizures, concerns about drug side effects, and uncertainty about their child's developmental trajectory. These chronic stressors may override the reassurance typically provided by satisfactory medical experiences, impairing the caregiver's ability to consistently support medication routines. Thus, the findings underscore the disruptive influence of caregiver anxiety in the context of epilepsy and highlight the need for integrated interventions that simultaneously address satisfaction with care and emotional resilience in caregivers [46].

### 8.3. Comparison With Previous Studies

Unlike prior studies that focused on the independent effects of social support or caregiver participation, this study is the first to construct a comprehensive model integrating social support, communication and decision-making participation, treatment satisfaction, and caregiver anxiety. It systematically reveals both the direct and indirect mechanisms by which these variables influence medication adherence. For instance, while previous studies mainly explored the direct effects of social support on adherence [19, 42, 43], this study further demonstrates that social support also exerts indirect effects through treatment satisfaction, a pathway rarely discussed in existing literature. Similarly, this study validates the direct and indirect effects of caregiver communication and decision-making participation through treatment satisfaction, providing valuable insights into caregiver involvement.

Additionally, the study identifies caregiver anxiety as a critical moderating variable, an area that has received limited attention in prior research. At high levels of anxiety, the positive effects of social support and communication and decision-making participation on adherence are significantly weakened. This indicates that these factors are less effective in improving adherence under conditions of high anxiety. Moreover, high anxiety also reduces the positive effect of treatment satisfaction on adherence, possibly due to distrust in treatment plans and excessive worry caused by anxiety. Even when caregivers report satisfaction with medical services, anxiety may lower their willingness to follow treatment plans, negatively affecting adherence. In pediatric epilepsy, the unpredictable nature of seizures and concerns about the child's development often lead to persistent feelings of helplessness and fear. Qualitative studies have shown that severe anxiety can cause emotional exhaustion, decision-making difficulties, and impaired processing of medical information, which undermine consistent adherence to treatment [47]. While some caregivers may become more vigilant, others may exhibit avoidant or inconsistent behaviors, especially under heavy emotional strain. These findings expand the understanding of the relationship between anxiety and adherence and provide new perspectives on the negative role of emotional variables in chronic disease management.

### 8.4. Clinical Implications

The findings of this study have important clinical implications for optimizing the management of pediatric epilepsy in China. First, caregiver social support plays a particularly vital role in epilepsy care. Given the unpredictable nature of seizures, the need for long-term medication adherence, and the strong stigma surrounding epilepsy in Chinese society, primary caregivers often experience significant psychological stress and social isolation [48, 49]. It is therefore essential to establish support systems specifically for families of children with epilepsy, such as peer support groups, online consultation platforms, and community-based mental health services, to reduce caregiver burden and enhance their caregiving capacity, thereby improving medication adherence [50, 51]. Second, caregiver participation in treatment decisions is especially critical in epilepsy management. Treatment often involves long-term adjustment of antiepileptic drugs, requiring informed and sustained cooperation from families. However, within the Chinese healthcare system, limited doctor-patient communication time often leaves caregivers in a passive role, undermining trust and adherence. Strengthening shared decision-making mechanisms, through respectful engagement with caregiver concerns, clear explanations of treatment goals, and discussion of potential side effects, can foster better adherence and clinical outcomes [44, 45]. Third, the frequent seizures, potential side effects of medication, and academic challenges commonly experienced by children with epilepsy increase caregiver anxiety and burden [6, 52]. Our findings indicate that treatment satisfaction significantly mediates medication adherence. Therefore, clinical services should be optimized to provide not only effective medical care but also empathetic, continuous support through regular follow-ups, individualized drug plans, and educational counseling, to enhance caregivers' confidence in managing the condition. Finally, psychological interventions for highly anxious caregivers are essential [34]. Pediatric epilepsy is often comorbid with cognitive or behavioral issues, which further compound caregiving challenges [1, 5]. In China, psychological resources for caregivers remain limited. Clinical teams should proactively identify high-risk caregivers and offer psychological assessments, group counseling, or online mental health interventions to help them manage emotional distress and strengthen coping strategies, ultimately contributing to better treatment adherence and long-term outcomes in children with epilepsy.

### 8.5. Limitations

Despite revealing the complex mechanisms by which social support, communication and decision-making participation, treatment satisfaction, and caregiver anxiety influence medication adherence, this study has certain limitations. First, its cross-sectional design limits the ability to infer causal relationships. Future research should adopt longitudinal designs to explore the dynamic causal relationships among variables. Second, this study did not include other psychological variables, such as depression or resilience, which may also influence adherence pathways. Future research should expand the scope of psychological variables examined. Third, the sample was drawn from medical institutions in Sichuan Province, which introduces some regional and population bias. Future studies should validate the generalizability of these findings in diverse cultural contexts. Fourth, the age range of participants (0–18 years) was relatively broad; future research should consider conducting subgroup analyses to account for developmental differences in adherence-related factors. Last, the measurement of adherence could only reflect parents' performance over a short time; a longitudinal study is needed to monitor parents' adherence in the longer term.

## 9. Conclusion

This study developed an integrated model to systematically examine the direct and indirect effects of caregiver social support, communication and decision-making participation, treatment satisfaction, and caregiver anxiety on medication adherence in pediatric epilepsy patients. Social support and caregiver communication not only directly promote adherence but also exert indirect effects through treatment satisfaction. Meanwhile, caregiver anxiety significantly moderates the strength of these pathways. Enhancing social support and caregiver communication, optimizing treatment satisfaction, and alleviating caregiver anxiety are effective strategies for improving medication adherence. These findings provide a theoretical basis for designing multilevel intervention measures and offer valuable directions for future research.

## Figures and Tables

**Table 1:** The basic information of included participants.

Variables	*n*	%
Gender		
Male	546	51.7
Female	510	48.3
Newly diagnosed		
Yes	533	50.5
No	523	49.5
Family history of epilepsy		
Yes	75	7.1
No	981	92.9
Seizures type		
Generalized epilepsy	405	38.4
Focal epilepsy	484	45.8
Generalized and focal epilepsy	123	11.6
Others	44	4.1
Only-child		
Yes	464	43.9
No	592	56.1%
Caregiver		
Parents	1,040	98.5
Nonparents	16	1.5
Caregivers' age		
<30 years	363	15.5
31–44 years	1711	73.3
45–59 years	237	10.2
>60 years	23	1.0
Caregivers' occupation		
Employed	603	57.1
Unemployed	453	42.9
CDMS	30.552 ± 6.86	
ANX	5.666 ± 6.386	
ADH	2.75 ± 1.691	
SAT	14.77 ± 3.649	
PSSS	52.907 ± 14.974	

*Note:* ADH, adherence to medication scale; ANX, generalized anxiety disorder; SAT, treatment satisfaction scale.

Abbreviations: CDMS, communication and decision-making scale; PSSS, perceived social support scale.

**Table 2:** Path analysis results.

Path analysis			Estimate	S.E.	C.R.	*p*	STD estimate
SAT	<---	PSSS	0.092	0.008	11.099	*⁣* ^*∗∗∗*^	0.483
SAT	<---	CDMS	0.072	0.013	5.47	*⁣* ^*∗∗∗*^	0.203
ADH	<---	CDMS	0.069	0.007	10.332	*⁣* ^*∗∗∗*^	0.392
ADH	<---	PSSS	0.032	0.004	7.894	*⁣* ^*∗∗∗*^	0.344
ADH	<---	SAT	0.044	0.018	2.425	0.015	0.09

*Note:* ADH, adherence to medication scale; ANX, generalized anxiety disorder; SAT, treatment satisfaction scale.

Abbreviations: CDMS, communication and decision-making scale; PSSS, perceived social support scale.

*⁣* ^*∗∗∗*^ *p* < 0.001.

**Table 3:** Mediation effect test.

Effect types	Path	Estimate	Lower	Upper	*p*
Direct effect	PSSS → ADH	0.032	0.024	0.041	<0.001
	CDMS → ADH	0.069	0.054	0.084	<0.001

Indirect effect	PSSS → SAT → ADH	0.004	0.001	0.008	0.018
	CDMS → SAT → ADH	0.003	0.001	0.007	0.018

Total effect	PSSS → ADH	0.036	0.029	0.044	<0.001
	CDMS → ADH	0.072	0.057	0.087	<0.001

*Note:* ADH, adherence to medication scale; ANX, generalized anxiety disorder; SAT, treatment satisfaction scale.

Abbreviations: CDMS, communication and decision-making scale; PSSS, perceived social support scale.

**Table 4:** Analysis of anxiety as a moderator in the relationship between PASS and ADH.

Variables	*B*	Standard error (SE)	*t*	*p*
Constant	2.924	0.261	11.220	<0.001*⁣*^*∗∗*^
Age	−0.011	0.012	−0.956	0.339
Whether the child is an only child	−0.109	0.093	−1.167	0.243
Caregiver's occupation	−0.043	0.094	−0.454	0.65
Comorbidities	0.266	0.093	2.854	0.004*⁣*^*∗∗*^
PSSS	0.05	0.003	15.969	<0.001*⁣*^*∗∗*^
ANX	−0.012	0.007	−1.625	0.105
PSSS*⁣*^*∗*^ ANX	−0.003	0.0005	−5.591	<0.001*⁣*^*∗∗*^
*R* ^2^	0.232			
*F*	*F* = 45.248, *p*=0.000			

*Note:* Dependent variable = total adherence score. ADH, adherence to medication scale; ANX, generalized anxiety disorder; SAT, treatment satisfaction scale.

Abbreviations: CDMS, communication and decision-making scale; PSSS, perceived social support scale.

*⁣* ^*∗*^ *p* < 0.05.

*⁣* ^*∗∗*^ *p* < 0.01.

**Table 5:** Analysis of anxiety as a moderator in the relationship between CDMS and ADH.

Variables	*B*	Standard error (SE)	*t*	*p*
Constant	2.985	0.259	11.527	<0.001*⁣*^*∗∗*^
Age	−0.008	0.012	−0.655	0.512
Whether the child is an only child	−0.104	0.093	−1.123	0.262
Caregiver's occupation	−0.064	0.094	−0.681	0.496
Comorbidities	0.151	0.093	1.619	0.106
CDMS	0.145	0.007	17.133	<0.001*⁣*^*∗∗*^
ANX	−0.011	0.007	−1.559	0.119
CDMS*⁣*^*∗*^ ANX	−0.005	0.001	−4.977	<0.001*⁣*^*∗∗*^
*R* ^2^	0.241			
*F*	*F* = 47.538, *p*=0.000			

*Note:* Dependent variable = total adherence score. ADH, adherence to medication scale; ANX, generalized anxiety disorder; SAT, treatment satisfaction scale.

Abbreviations: CDMS, communication and decision-making scale; PSSS, perceived social support scale.

*⁣* ^*∗*^ *p* < 0.05.

*⁣* ^*∗∗*^ *p* < 0.01.

**Table 6:** Analysis of anxiety as a moderator in the relationship between SAT and ADH.

Variables	*B*	Standard error (SE)	*t*	*p*
Constant	2.999	0.268	11.186	<0.001*⁣*^*∗∗*^
Age	−0.017	0.012	−1.406	0.16
Whether the child is an only child	−0.072	0.096	−0.751	0.453
Caregiver's occupation	−0.105	0.097	−1.085	0.279
Comorbidities	0.281	0.096	2.927	0.004*⁣*^*∗∗*^
SAT	0.179	0.013	13.762	<0.001*⁣*^*∗∗*^
ANX	−0.009	0.007	−1.213	0.225
SAT*⁣*^*∗*^ ANX	−0.01	0.002	−5.261	<0.001*⁣*^*∗∗*^
*R* ^2^	0.186			
*F*	*F* = 34.158, *p*=0.000			

*Note:* Dependent variable = total adherence score. ADH, adherence to medication scale; ANX, generalized anxiety disorder; SAT, treatment satisfaction scale.

Abbreviations: CDMS, communication and decision-making scale; PSSS, perceived social support scale.

*⁣* ^*∗*^ *p* < 0.05.

*⁣* ^*∗∗*^ *p* < 0.01.

**Table 7:** Cronbach reliability analysis.

Measurement content	Variables	Corrected item-total correlation (CITC)	Cronbach's alpha if item deleted	Cronbach's alpha
ADH	ADH1	0.794	0.927	0.932
	ADH2	0.858	0.906	
	ADH3	0.853	0.907	
	ADH4	0.857	0.906	

SAT	SAT1	0.731	0.893	0.903
	SAT2	0.811	0.865	
	SAT3	0.845	0.852	
	SAT4	0.748	0.888	

CDMS: information sharing and transparency	PCDMS1	0.774	0.809	0.874
	PCDMS5	0.762	0.819	
	PCDMS9	0.739	0.84	

CDMS: decision-making participation and consultation	PCDMS2	0.687	0.771	0.831
	PCDMS3	0.647	0.81	
	PCDMS4	0.74	0.717	

CDMS: personalization and patient care	PCDMS6	0.738	0.752	0.843
	PCDMS7	0.747	0.742	
	PCDMS8	0.643	0.84	

PSSS: others' support	PSSS1	0.79	0.893	0.914
	PSSS2	0.814	0.885	
	PSSS5	0.8	0.89	
	PSSS10	0.813	0.885	

PSSS: family support	PSSS3	0.806	0.882	0.911
	PSSS4	0.828	0.874	
	PSSS8	0.776	0.892	
	PSSS11	0.782	0.89	

PSSS: friend support	PSSS6	0.848	0.907	0.931
	PSSS7	0.832	0.912	
	PSSS9	0.846	0.908	
	PSSS12	0.827	0.914	

ANX	ANX1	0.823	0.932	0.942
	ANX2	0.834	0.931	
	ANX3	0.835	0.931	
	ANX4	0.814	0.933	
	ANX5	0.793	0.935	
	ANX6	0.761	0.938	
	ANX7	0.804	0.934	

**Table 8:** KMO and Bartlett's test.

KMO		0.915
Bartlett's Test of sphericity	Approximate chi-square	28,634.016
	*df*	630
	*p*-Value	<0.001

**Table 9:** Table of variance explained.

Factor number	Eigenvalue			Variance explained before rotation			Variance explained after rotation		
	Eigenvalue	Variance explained (%)	Cumulative (%)	Eigenvalue	Variance explained (%)	Cumulative (%)	Eigenvalue	Variance explained (%)	Cumulative (%)
1	9.657	26.824	26.824	9.657	26.824	26.824	5.229	14.526	14.526
2	5.194	14.426	41.25	5.194	14.426	41.25	3.333	9.257	23.783
3	3.908	10.857	52.107	3.908	10.857	52.107	3.29	9.138	32.922
4	2.038	5.662	57.769	2.038	5.662	57.769	3.216	8.933	41.855
5	1.901	5.279	63.048	1.901	5.279	63.048	3.172	8.81	50.665
6	1.793	4.981	68.03	1.793	4.981	68.03	3.157	8.769	59.434
7	1.52	4.222	72.252	1.52	4.222	72.252	2.401	6.669	66.103
8	1.258	3.495	75.747	1.258	3.495	75.747	2.314	6.429	72.532
9	1.069	2.969	78.716	1.069	2.969	78.716	2.227	6.185	78.716
10	0.489	1.358	80.075	—	—	—	—	—	—
11	0.464	1.288	81.363	—	—	—	—	—	—
12	0.401	1.113	82.476	—	—	—	—	—	—
13	0.385	1.069	83.545	—	—	—	—	—	—
14	0.374	1.039	84.584	—	—	—	—	—	—
15	0.367	1.018	85.602	—	—	—	—	—	—
16	0.352	0.977	86.579	—	—	—	—	—	—
17	0.334	0.927	87.506	—	—	—	—	—	—
18	0.315	0.876	88.381	—	—	—	—	—	—
19	0.31	0.86	89.241	—	—	—	—	—	—
20	0.301	0.835	90.076	—	—	—	—	—	—
21	0.294	0.816	90.892	—	—	—	—	—	—
22	0.286	0.794	91.687	—	—	—	—	—	—
23	0.277	0.771	92.457	—	—	—	—	—	—
24	0.272	0.756	93.213	—	—	—	—	—	—
25	0.257	0.713	93.926	—	—	—	—	—	—
26	0.247	0.685	94.61	—	—	—	—	—	—
27	0.241	0.671	95.281	—	—	—	—	—	—
28	0.229	0.635	95.916	—	—	—	—	—	—
29	0.219	0.607	96.524	—	—	—	—	—	—
30	0.217	0.602	97.125	—	—	—	—	—	—
31	0.208	0.578	97.703	—	—	—	—	—	—
32	0.197	0.547	98.25	—	—	—	—	—	—
33	0.182	0.506	98.756	—	—	—	—	—	—
34	0.17	0.473	99.229	—	—	—	—	—	—
35	0.151	0.421	99.65	—	—	—	—	—	—
36	0.126	0.35	100	—	—	—	—	—	—

**Table 10:** Table of rotated factor loadings.

Variables	Factor loadings coefficient								
	Factor 1	Factor 2	Factor 3	Factor 4	Factor 5	Factor 6	Factor 7	Factor 8	Factor 9
ADH1	−0.028	**0.813**	0.151	0.121	0.126	0.127	0.122	0.101	0.143
ADH2	−0.022	**0.856**	0.118	0.129	0.129	0.124	0.136	0.112	0.159
ADH3	−0.018	**0.853**	0.129	0.109	0.135	0.175	0.128	0.101	0.124
ADH4	−0.043	**0.856**	0.131	0.11	0.149	0.161	0.106	0.133	0.111
SAT1	−0.095	0.088	0.122	0.147	0.16	**0.792**	0.028	0.069	0.099
SAT2	−0.019	0.145	0.144	0.13	0.107	**0.85**	0.089	0.077	0.048
SAT3	−0.023	0.171	0.151	0.154	0.113	**0.856**	0.099	0.07	0.093
SAT4	−0.021	0.138	0.113	0.102	0.119	**0.823**	0.041	0.059	0.051
CDMS1	−0.005	0.123	0.048	0.074	0.051	0.102	**0.836**	0.215	0.191
CDMS5	−0.003	0.136	0.041	0.041	0.058	0.11	**0.827**	0.228	0.18
CDMS9	0.029	0.197	0.055	0.078	0.066	0.03	**0.799**	0.212	0.2
CDMS2	−0.05	0.1	0.048	0.054	0.075	0.073	0.261	0.24	**0.768**
CDMS3	−0.014	0.19	0.048	0.019	0.005	0.074	0.112	0.073	**0.827**
CDMS4	−0.039	0.188	0.018	0.054	0.059	0.131	0.21	0.185	**0.807**
CDMS6	−0.061	0.154	0.076	0.073	0.053	0.068	0.257	**0.799**	0.177
CDMS7	−0.024	0.157	0.061	0.038	0.038	0.122	0.212	**0.822**	0.162
CDMS8	0.043	0.085	0.02	0.004	0.051	0.066	0.16	**0.816**	0.121
PSSS1	−0.019	0.149	0.184	0.151	**0.824**	0.153	0.007	0.041	0.055
PSSS2	−0.046	0.098	0.202	0.197	**0.833**	0.137	0.064	0.051	0.019
PSSS5	−0.02	0.136	0.21	0.174	**0.822**	0.126	0.068	0.037	0.042
PSSS10	−0.014	0.147	0.266	0.18	**0.813**	0.114	0.056	0.039	0.034
PSSS3	−0.019	0.101	0.155	**0.848**	0.145	0.149	0.042	0.055	0.031
PSSS4	−0.014	0.109	0.166	**0.863**	0.157	0.113	0.059	0.023	0.03
PSSS8	0.001	0.093	0.167	**0.824**	0.171	0.136	0.025	0.008	0.048
PSSS11	0.016	0.131	0.173	**0.815**	0.184	0.131	0.074	0.036	0.025
PSSS6	−0.03	0.124	**0.845**	0.174	0.224	0.159	0.023	0.043	0.015
PSSS7	0.011	0.137	**0.843**	0.154	0.222	0.146	0.016	0.019	0.031
PSSS9	0.009	0.121	**0.838**	0.217	0.203	0.15	0.076	0.078	0.021
PSSS12	−0.005	0.145	**0.835**	0.174	0.22	0.117	0.05	0.041	0.069
ANX1	**0.873**	−0.002	−0.004	0.006	−0.012	−0.012	0.03	−0.006	0.001
ANX2	**0.88**	−0.013	−0.032	−0.005	−0.014	−0.007	0.019	−0.026	−0.056
ANX3	**0.883**	−0.003	−0.025	−0.023	−0.031	−0.035	−0.033	−0.01	−0.002
ANX4	**0.866**	−0.02	−0.009	0.011	−0.007	−0.016	0.003	−0.009	−0.016
ANX5	**0.849**	0.001	0.013	0.007	−0.042	−0.028	0.009	−0.005	−0.008
ANX6	**0.824**	−0.053	0.015	−0.009	−0.002	0.008	0.008	0.033	0.019
ANX7	**0.857**	−0.018	0.018	−0.011	0.01	−0.071	−0.031	−0.017	−0.045

*Note:* Rotation method: varimax (maximum variance method). Bold represents belonging to a dimension.

**Table 11:** Table of model fit indices.

Indicators	CMIN/DF	RMR	GFI	NFI	TLI	CFI	RMSEA
Optimal values	<3	<0.05	>0.9	>0.9	>0.9	>0.9	<0.08
Acceptable values	<5	<0.08	>0.8	>0.8	>0.8	>0.8	<0.1
Measured results	1.971	0.034	0.945	0.962	0.978	0.981	0.030

**Table 12:** Factor loading table.

Items			Estimate	S.E.	C.R.	*p*	STD estimate
ADH1	<---	ADH	1	—	—	—	0.821
ADH2	<---	ADH	1.075	0.03	35.596	*⁣* ^*∗∗∗*^	0.886
ADH3	<---	ADH	1.09	0.03	36.599	*⁣* ^*∗∗∗*^	0.902
ADH4	<---	ADH	1.096	0.029	37.173	*⁣* ^*∗∗∗*^	0.911
SAT1	<---	SAT	1	—	—	—	0.771
SAT2	<---	SAT	1.186	0.039	30.728	*⁣* ^*∗∗∗*^	0.875
SAT3	<---	SAT	1.238	0.038	32.333	*⁣* ^*∗∗∗*^	0.92
SAT4	<---	SAT	1.056	0.039	26.844	*⁣* ^*∗∗∗*^	0.782
CDMS1	<---	Information sharing and transparency	1	—	—	—	0.852
CDMS5	<---	Information sharing and transparency	1.008	0.032	31.413	*⁣* ^*∗∗∗*^	0.842
CDMS9	<---	Information sharing and transparency	0.973	0.032	30.275	*⁣* ^*∗∗∗*^	0.815
CDMS2	<---	Decision-making participation and consultation	1	—	—	—	0.798
CDMS3	<---	Decision-making participation and consultation	0.917	0.04	23.094	*⁣* ^*∗∗∗*^	0.711
CDMS4	<---	Decision-making participation and consultation	1.081	0.04	26.958	*⁣* ^*∗∗∗*^	0.861
CDMS6	<---	Personalization and patient care	1	—	—	—	0.855
CDMS7	<---	Personalization and patient care	0.964	0.033	29.49	*⁣* ^*∗∗∗*^	0.849
CDMS8	<---	Personalization and patient care	0.752	0.031	24.112	*⁣* ^*∗∗∗*^	0.7
PSSS1	<---	Others' support	1	—	—	—	0.835
PSSS2	<---	Others' support	1.064	0.031	34.112	*⁣* ^*∗∗∗*^	0.861
PSSS5	<---	Others' support	1.116	0.033	33.396	*⁣* ^*∗∗∗*^	0.849
PSSS10	<---	Others' support	1.075	0.031	34.417	*⁣* ^*∗∗∗*^	0.866
PSSS3	<---	Family support	1	—	—	—	0.858
PSSS4	<---	Family support	1.046	0.028	36.832	*⁣* ^*∗∗∗*^	0.882
PSSS8	<---	Family support	0.946	0.029	33.031	*⁣* ^*∗∗∗*^	0.823
PSSS11	<---	Family support	0.978	0.029	33.536	*⁣* ^*∗∗∗*^	0.831
PSSS6	<---	Friend support	1	—	—	—	0.889
PSSS7	<---	Friend support	0.966	0.024	39.946	*⁣* ^*∗∗∗*^	0.871
PSSS9	<---	Friend support	1.002	0.024	41.536	*⁣* ^*∗∗∗*^	0.888
PSSS12	<---	Friend support	0.983	0.025	39.503	*⁣* ^*∗∗∗*^	0.866
ANX1	<---	ANX	1	—	—	—	0.848
ANX2	<---	ANX	1.047	0.029	36.487	*⁣* ^*∗∗∗*^	0.864
ANX3	<---	ANX	1.053	0.029	36.726	*⁣* ^*∗∗∗*^	0.868
ANX4	<---	ANX	1.008	0.029	35.029	*⁣* ^*∗∗∗*^	0.844
ANX5	<---	ANX	0.977	0.029	33.268	*⁣* ^*∗∗∗*^	0.819
ANX6	<---	ANX	0.965	0.031	31.086	*⁣* ^*∗∗∗*^	0.785
ANX7	<---	ANX	0.97	0.028	34.269	*⁣* ^*∗∗∗*^	0.833

*⁣* ^*∗∗∗*^ *p* < 0.01.

**Table 13:** Convergent validity.

Dimension	CR	AVE
ADH	0.933	0.776
SAT	0.904	0.704
CDMS1: information sharing and transparency	0.875	0.7
CDMS2: decision-making participation and consultation	0.834	0.628
CDMS3: personalization and patient care	0.845	0.647
PSSS1: others' support	0.914	0.728
PSSS2: family support	0.912	0.721
PSSS3: friend support	0.931	0.772
ANX	0.943	0.702

**Table 14:** Discriminant validity.

Variables	ADH	SAT	CDMS1	CDMS2	CDMS3	PSSS1	PSSS2	PSSS3	ANX
ADH	0.881	—	—	—	—	—	—	—	—
SAT	0.428*⁣*^*∗∗∗*^	0.839	—	—	—	—	—	—	—
CDMS1	0.417*⁣*^*∗∗∗*^	0.285*⁣*^*∗∗∗*^	0.837	—	—	—	—	—	—
CDMS2	0.446*⁣*^*∗∗∗*^	0.308*⁣*^*∗∗∗*^	0.586*⁣*^*∗∗∗*^	0.792	—	—	—	—	—
CDMS3	0.399*⁣*^*∗∗∗*^	0.284*⁣*^*∗∗∗*^	0.616*⁣*^*∗∗∗*^	0.531*⁣*^*∗∗∗*^	0.805	—	—	—	—
PSSS1	0.407*⁣*^*∗∗∗*^	0.397*⁣*^*∗∗∗*^	0.220*⁣*^*∗∗∗*^	0.203*⁣*^*∗∗∗*^	0.203*⁣*^*∗∗∗*^	0.853	—	—	—
PSSS2	0.352*⁣*^*∗∗∗*^	0.397*⁣*^*∗∗∗*^	0.215*⁣*^*∗∗∗*^	0.183*⁣*^*∗∗∗*^	0.177*⁣*^*∗∗∗*^	0.487*⁣*^*∗∗∗*^	0.849	—	—
PSSS3	0.395*⁣*^*∗∗∗*^	0.416*⁣*^*∗∗∗*^	0.199*⁣*^*∗∗∗*^	0.180*⁣*^*∗∗∗*^	0.209*⁣*^*∗∗∗*^	0.579*⁣*^*∗∗∗*^	0.485*⁣*^*∗∗∗*^	0.879	—
ANX	−0.062	−0.075*⁣*^*∗*^	−0.008	−0.077*⁣*^*∗*^	−0.053	−0.058	−0.024	−0.025	0.838

*⁣* ^*∗*^ *p* < 0.05.

*⁣* ^*∗∗∗*^ *p* < 0.001.

**Table 15:** Correlation analysis.

Variables	Mean	Standard deviation	ADH	SAT	PSSS	CDMS	ANX
ADH	2.75	1.691	1	—	—	—	—
SAT	14.77	3.649	0.390*⁣*^*∗∗*^	1	—	—	—
PSSS	52.907	14.974	0.446*⁣*^*∗∗*^	0.463*⁣*^*∗∗*^	1	—	—
CDMS	30.552	6.86	0.465*⁣*^*∗∗*^	0.307*⁣*^*∗∗*^	0.266*⁣*^*∗∗*^	1	—
ANX	5.666	6.386	−0.058	−0.077*⁣*^*∗*^	−0.037	−0.041	1

*⁣* ^*∗*^ *p*  < 0.05.

*⁣* ^*∗∗*^ *p*  < 0.01.

**Table 16:** Structural equation model fit index analysis.

Indicator	CMIN/DF	RMR	GFI	NFI	TLI	CFI	RMSEA
Optimal indicator	<3	<0.05	>0.9	>0.9	>0.9	>0.9	<0.08
Acceptable l indicator	<5	<0.08	>0.8	>0.8	>0.8	>0.8	<0.1
Measurement result	3.205	0.102	0.971	0.974	0.977	0.982	0.046

## Data Availability

The data that support the findings of this study are available from the corresponding author upon reasonable request.
